# Constitutive Expression of the Immunosuppressive Tryptophan Dioxygenase TDO2 in Glioblastoma Is Driven by the Transcription Factor C/EBPβ

**DOI:** 10.3389/fimmu.2020.00657

**Published:** 2020-05-14

**Authors:** Takumi Kudo, Mirja T. Prentzell, Soumya R. Mohapatra, Felix Sahm, Zhongliang Zhao, Ingrid Grummt, Wolfgang Wick, Christiane A. Opitz, Michael Platten, Edward W. Green

**Affiliations:** ^1^DKTK CCU Neuroimmunology and Brain Tumor Immunology, German Cancer Research Center (DKFZ), Heidelberg, Germany; ^2^Department of Neurology, Medical Faculty Mannheim, Heidelberg University, Mannheim, Germany; ^3^Department of Neurosurgery, Tokyo Medical and Dental University, Tokyo, Japan; ^4^DKTK Brain Cancer Metabolism Group, German Cancer Research Center (DKFZ), Heidelberg, Germany; ^5^Department of Neuropathology, Institute of Pathology, University Hospital Heidelberg, Heidelberg, Germany; ^6^DKTK Division Molecular Biology of the Cell, German Cancer Research Center (DKFZ), Heidelberg, Germany; ^7^DKTK CCU Neurooncology, German Cancer Research Center (DKFZ), Heidelberg, Germany; ^8^Neurology Clinic and National Center for Tumor Diseases, University Hospital of Heidelberg, Heidelberg, Germany

**Keywords:** TDO2, glioblastoma, regulation, CEBPB, tryptophan, IL1B

## Abstract

Catabolism of the essential amino acid tryptophan is a key metabolic pathway contributing to the immunosuppressive tumor microenvironment and therefore a viable drug target for cancer immunotherapy. In addition to the rate-limiting enzyme indoleamine-2,3-dioxygenase-1 (IDO1), tryptophan catabolism via tryptophan-2,3-dioxygenase (TDO2) is a feature of many tumors, particularly malignant gliomas. The pathways regulating TDO2 in tumors are poorly understood; using unbiased promoter and gene expression analyses, we identify a distinct CCAAT/enhancer-binding protein β (C/EBPβ) binding site in the promoter of TDO2 essential for driving constitutive TDO2 expression in glioblastoma cells. Using The Cancer Genome Atlas (TCGA) data, we find that C/EBPβ expression is correlated with TDO2, and both are enriched in malignant glioma of the mesenchymal subtype and associated with poor patient outcome. We determine that TDO2 expression is sustained mainly by the LAP isoform of CEBPB and interleukin-1β, which activates TDO2 via C/EBPβ in a mitogen-activated protein kinase (MAPK) kinase-dependent fashion. In summary, we provide evidence for a novel regulatory and therapeutically targetable pathway of immunosuppressive tryptophan degradation in a subtype of glioblastoma with a particularly poor prognosis.

## Introduction

Malignant gliomas are characterized by profound local and systemic immunosuppression (1), which blunts the efficacy of the novel immunotherapeutic treatments for malignant gliomas (2). Overcoming the immunosuppressive glioma microenvironment is therefore an important challenge (3). The enzymatic degradation of tryptophan (Trp) to kynurenine (Kyn), mediated by indoleamine-2,3-dioxygenase-1 (IDO1) or tryptophan-2,3-dioxygenase (TDO2), is a key immunosuppressive pathway operative in gliomas and other types of tumors (4–8), and an emerging drug target in cancer immunotherapy (9–11). Trp metabolism promotes local immune tolerance by both inducing anergy and apoptosis of CD8-positive T cells, and activating regulatory T (Treg) cells, mainly through accumulation of immunosuppressive Kyn (12, 13). IDO1 is known to be overexpressed in many tumors – including gliomas – with both tumor and stromal cells contributing to IDO1 activity (5, 14–16). More recently, TDO2 has been shown to be expressed in many tumors and to promote tumor growth by suppressing antitumor immune responses in a similar fashion (17–20) (Supplementary Figure S1). In contrast to IDO1, whose regulation has been thoroughly characterized and involves tumor suppressor genes (21), oncogenes (22), growth factor/cytokine signaling pathways (23), and epigenetic mechanisms (24), the regulation of TDO2 in gliomas is not well understood. Here, we employed an unbiased approach to identify transcriptional regulators of TDO2.

## Materials and Methods

### Cell Culture

The human malignant glioma cell line T98G was obtained from the American Type Culture Collection (ATCC), and cultured in Dulbecco’s modified Eagle’s medium (DMEM) containing 10% fetal bovine serum (Gibco), penicillin (100 U/mL), and streptomycin (100 μg/mL) (Gibco). Interleukin-1β (IL-1β) was purchased from R&D Systems (201-LB-005); SB203580 was purchased from AdipoGen (AG-CR1-0030-M001).

### TDO2 Expression, Reporter Cloning and Luciferase Experiments

Fragments of the human TDO2 enhancer were amplified from T98G cells using the primers listed in Supplementary Table S1 and ligated into the minimal promoter vector pGL4.26[luc2/minP/Hygro] (Promega E8441) using *BglII–XhoI* restriction sites. Reporter constructs were transfected into T98G cells using FuGene HD (Promega E2311), and cells were simultaneously co-transfected with a constitutively active renilla luciferase-expressing plasmid (pRL-TK, Promega E2231) as a transfection control. Forty-eight hours after transfection, reporter assays were performed according to the manufacturers’ protocol using the Promega Dual-Luciferase Reporter Assay System (Promega E1910) and a PHERAstar FS instrument (BMG Labtech). Firefly luciferase was normalized to renilla luciferase expression. The CEBPB consensus sequence deletion construct was made using the Q5^®^ Site-Directed Mutagenesis Kit (New England Biolabs E0554S) using primers listed in Supplementary Table S1.

### Enhancer Binding Site Analysis

The enhancer region of TDO2 (−130 to −92 bp) was screened for putative transcription factor binding sites using an online implementation of TFBIND^1^. TFBIND identifies putative transcription factor binding sites by identifying regions similar to those of transcription factor consensus binding motifs, using transcription factor-specific similarity cutoffs derived from the TRANSFAC database (R3.4). The TRANSFAC consensus motif identified for CEBPB in the TDO2 promoter was V$CEBPB_01, consensus motif “*RNRTKNNGMAAKNN*” with a score of 0.84 (exceeding the cutoff score of 0.81).

### Chromatin Immunoprecipitation

Cells were fixed by adding formaldehyde directly to culture medium for 10 min (final concentration 1.0%). The reaction was quenched using glycine (final concentration 125 mM). Cells were washed twice with phosphate-buffered saline (PBS) before being resuspended in buffer A [100 mM of Tris-HCl at pH 8.0, and 10 mM of dithiothreitol (DTT)] on ice for 15 min, followed by vortexing and incubation at 30°C for 15 min. Cells were then spun down and resuspended iteratively in buffer B [10 mM of HEPES at pH 7.5, 10 mM of EDTA, 0.5 mM of EGTA, and 0.25% (w/v) Triton X-100], buffer C (10 mM of HEPES at pH 7.5, 10 mM of EDTA, 0.5 mM of EGTA, and 200 mM of NaCl), and finally buffer D [50 mM of Tris-HCl at pH 8.0, 10 mM of EDTA, 1% sodium dodecyl sulfate (SDS), and 0.5% proteinase inhibitor cocktail]. Samples were sonicated for 10 min, spun down, and sonicated for a further 10 min to generate fragments of 300–500 bp of length. DNA fragments were spun down and resuspended 1:5 with immunoprecipitation (IP) buffer [15 mM of Tris-HCl at pH 8.0, 1.2 mM of EDTA, 180 mM of NaCl, and 1.2% (w/v) Triton X-100]. Ten μL of blocked Protein A/G sepharose beads were added per mL of IP buffer, and the samples were incubated at 4°C for 1 h to pre-clear chromatin. One mL of supernatant was then incubated overnight at 4°C with 1–3 μg of antibody. A further 10 μL of blocked Protein A/G sepharose beads was added to each sample and incubated at 4°C for 1 h. Beads were then washed twice with 1 mL of each of the following buffers: low salt wash buffer [20 mM of Tris-HCl at pH 8.0, 2 mM of EDTA, 150 mM of NaCl, 0.1% (w/v) SDS, 1% (w/v) Triton X-100], High salt wash buffer (20 mM of Tris-HCl at pH 8.0, 2 mM of EDTA, 500 mM of NaCl, 0.1% (w/v) SDS, and 1% (w/v) Triton X-100), LiCl wash buffer [10 mM of Tris-HCl at pH 8.0, 1 mM of EDTA, 250 mM of LiCl, 1% (w/v) NP40, and 1% (w/v) deoxycholate], and Tris-EDTA (TE) buffer (pH 8.0). One hundred μL of 10% slurry was added to the beads. Samples were then boiled for 10 min, followed by 30 min incubation at 52°C with 40 μg of Protein K, and a further 10 min boiling. Beads were spun down, and the supernatant containing the precipitated DNA was analyzed by PCR using the primers in Supplementary Table S1.

### CEBPB Overexpression

A custom codon-optimized cDNA of CEBPB in pDONR221 was purchased from Thermo Fisher Scientific (full sequence listed in Supplementary Table S1). LAP and LIP isoforms of CEBPB were generated by site-directed mutagenesis (New England Biolabs E0554S) using primers listed in Supplementary Table S1. Isoforms were cloned into the pMXs-GW-IRES-puro destination vector using Gateway^TM^ cloning technology (Invitrogen). Cells were transfected using FuGene HD following the manufacturer’s protocols (Promega). Expression values were compared using two-way ANOVA with Tukey’s correction for multiple comparisons testing.

### Bioinformatic Analysis of the Cancer Genome Atlas (TCGA) data, Survival Curves, and Gene Association

Expression data, overall survival data, and Spearman’s correlation coefficients for glioblastoma (GBM) patients in The Cancer Genome Atlas (TCGA) study was downloaded from cBioportal and Firebrowse^3^. Rank product was calculated as geometric mean of each rank. Subtype-specific analyses were performed on TCGA data downloaded using Firebrowse^3^ followed by statistical analysis in R. For overall survival analysis, 525 GBM patients were divided into two groups according to CEBPB mRNA expression. *p*-value was calculated using the log-rank test in R.

### Immunohistochemistry

Formalin-fixed paraffin-embedded GBM tissue was obtained from the archives of the Department of Neuropathology, Institute of Pathology, Heidelberg. For immunohistochemistry, sections cut into 3 μm were incubated and processed with rabbit anti-human TDO antibody (25) and rabbit anti-human C/EBPβ antibody (C-19, Santa Cruz Biotechnology) on a Ventana BenchMark XT immunostainer (Ventana Medical Systems, Tucson, AZ, United States). The Ventana staining procedure included pre-treatment with cell conditioner 1 (pH 6.0) for 60 min and followed by antibody incubation at 37°C for 32 min. Incubation was followed by Ventana standard signal amplification, UltraWash, counter−staining with one drop of hematoxylin for 4 min and one drop of bluing reagent for 4 min. For visualization, ultraViewUniversal DAB Detection Kit (Ventana Medical Systems) was used. For quantification of C/EBPβ and TDO protein levels, histo-scores with relative expression levels were calculated, and correlation was assessed using Spearman’s rank correlation coefficient.

### Quantitative Reverse Transcription PCR

Total RNA was extracted with RNeasy mini kit (Qiagen) followed by cDNA synthesis using the Applied Biosystems reverse transcription kit (Thermo Fisher). mRNA from biological triplicate experiments was measured by quantitative reverse transcription (qRT) PCR using primaQUANT SYBR Green reagents (Steinbrenner Laborsysteme GmbH) on a LightCycler 480 instrument (Roche). The primers for qRT-PCR were CAAATCCTCTGGGAGTTGGA (human TDO2 Fw), GTCCAAGGCTGTCATCGTCT (human TDO2 Rv), AAGCACAGCGACGAGTACAA (human CEBPB Fw), GTGAGCTCCAGGACCTTGTG (human CEBPB Rv), CCCCGGTTTCTATAAATTGAGC (human GAPDH Fw), and CACCTTCCCCATGGTGTCT (human GAPDH Rv). Levels of TDO2 and CEBPB were calculated relative to GAPDH, with each reaction performed as a technical triplicate, and the results were normalized to TDO2 or CEBPB levels in respective control samples. Two-way ANOVA statistical analysis was performed using Prism 8.0, followed by Tukey’s multiple comparisons tests, where appropriate.

### Small Interfering RNA Experiments

Small interfering RNAs (siRNA) targeting CEBPB were ordered from Dharmacon (SMART-pool L-006423-00-0005), together with the ON-TARGET plus non-targeting pool control siRNAs (D-001810-10-05). siRNA target sequences were CCUCGCAGGUCAAGAGCAA, CUGCUU GGCUGCUGCGUAC, GCGCUUACCUCGGCUACCA, GCAC CCUGCGGAACUUGUU (human CEBPB) and UGGUUUAC AUGUCGACUAA, UGGUUUACAUGUUGUGUGA, UGGUU UACAUGUUUUCUGA, UGGUUUACAUGUUUUCCUA (non-targeting pool). Transfection of T98G cells was performed with Lipofectamine RNAiMAX (Invitrogen) according to the manufacturer’s protocol, and knockdown efficiency was verified by either qRT-PCR or Western blot.

### Western Blotting

Whole cell lysates were lysed with radioimmunoprecipitation assay (RIPA) buffer [50 mM/l of Tris-HCl (pH 8.0), 150 mM/l of sodium chloride, 0.5% (w/v) sodium deoxycholate, 0.1% (w/v) SDS, and 1.0% (w/v) NP-40 substitute] with complete protease inhibitor (Roche) for 20 min on ice and then centrifuged for 10 min (4°C, 13,000 rpm). Protein concentrations were measured by the Bio-Rad protein assay (Bio-Rad). Twenty μg of each samples was loaded on SDS–polyacrylamide gel electrophoresis (PAGE) and transferred to nitrocellulose membrane following immunoblotting using anti-C/EBPβ rabbit monoclonal (clone C-19, Santa Cruz) or anti-GAPDH goat polygclonal (LINARIS Biologische Produkte GmbH #LAH1064) antibodies. Pierce enhanced chemiluminescence (ECL) western blotting substrate was used to detect chemiluminescence using a ChemiDoc XRS + camera. Raw images taken were processed with Image Lab (version 5.2.1) and exported for publication as TIFF files with 600 dpi resolution. Quantification was performed using Image Lab; pixel values of each lane were normalized to the average value of all lanes and then normalized to the loading control GAPDH.

### Kynurenine High-Performance Liquid Chromatography

High-performance liquid chromatography (HPLC) measurement of Kyn was performed in cell culture supernatants collected from each treatment type. One mL of supernatant sample was first treated with 168.6 μL of 72% trichloroacetic acid to precipitate out dissolved proteins; subsequently, samples were centrifuged at maximum speed for 10 min, and the resulting protein-free supernatants were transferred into glass HPLC vials for further HPLC analysis. Chromatographic separation of Kyn was achieved on a Dionex Ultimate^®^ 3000 UHPLC (Thermo Scientific) on a reversed-phase Accucore^TM^ aQ column (Thermo Scientific^TM^) with 2.6-μm particle size. The mobile phase gradient consisted of 0.1% trifluoroacetic acid (TFA) in water (A) and 0.1% TFA in acetonitrile (B). Standards for all analytes were utilized to determine the retention time and UV emission spectrum at 365 nm for Kyn. Analyte concentration in samples was analyzed by comparison with the respective standards using the Chromeleon^TM^ 7.2 Chromatography Data System (Thermo Scientific^TM^ Dionex^TM^).

## Results

### Identification of the Enhancer Motif Essential for Constitutive TDO2 Expression in T98G Glioma Cells

**FIGURE 1:**
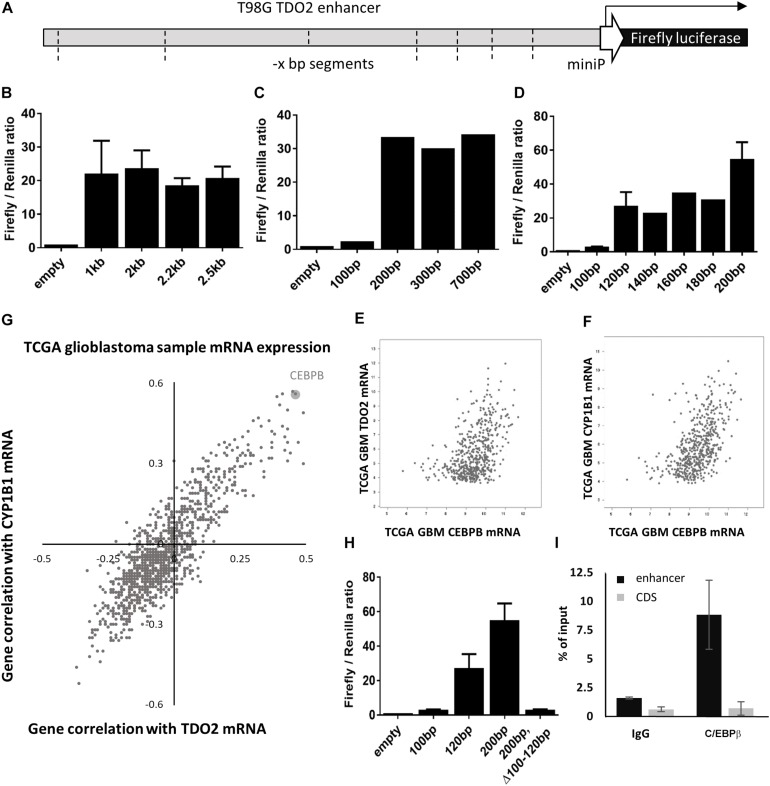
*In vitro* analysis of TDO2 enhancer. **(A)** Schematic of the pGL4.26 reporter containing 2.5 kb of the TDO2 enhancer driving expression of a firefly luciferase gene. **(B–D)** Iterative deletion of the TDO2 enhancer defines a region between −100 and −120 bp as essential for TDO2 expression. Values are the mean of technical triplicates; standard error bars represent repeat experiments [*n* = 2 for B, *n* = 3 for selected constructs in panel **(D)**]. **(E)** Spearman’s correlation coefficient was calculated between each gene expressed in The Cancer Genome Atlas (TCGA) glioblastoma (GBM) patients and both TDO2 and TDO2-induced aryl hydrocarbon receptor (AHR) target gene CYP1B1. **(F)** Correlation between CEBPB and TDO2 expression in TCGA GBM patients (Spearman: 0.47). **(G)** Correlation between CEBPB and CYP1B1 expression in TCGA GBM patients (Spearman: 0.56). **(H)** Deletion of the C/EBPβ binding site in the −100 to −120 bp enhancer region abrogates TDO2 expression. **(I)** Chromatin precipitation assay confirms an interaction between the TDO2 enhancer and C/EBPβ protein. Data are expressed ± standard deviation.

To identify the enhancer elements essential for the transcription of TDO2, 2.5 kb of enhancer sequence 5’ of the TDO2 transcriptional start site was cloned from the constitutively TDO2-expressing T98G glioma cell line (18, 26). Insertion of this sequence into the pGL4.26 firefly luciferase reporter vector and transfection of this vector into T98G cells was confirmed to result in luciferase expression (Figure 1A). Iterative deletion of regions of the TDO2 enhancer (Figures 1B–D) revealed that motifs located between −100 and −120 bp from the transcriptional start site were essential for complete activation of the reporter construct, while the region between −180 and −200 bp contributed to high levels of TDO2 expression.

We used the TFBIND database (27) to identify 21 transcription factors with putative binding sites that would be disrupted by a deletion in the essential −100 and −120 bp region (Table 1). To narrow down this list to transcription factors driving TDO2 expression in gliomas, we interrogated TCGA dataset to find transcription factors showing strong transcriptional correlations with TDO2 (Table 2). TDO2 protein catalyzes the conversion of Trp to Kyn, activating the aryl hydrocarbon receptor (AHR) and leading to the upregulation of AHR target genes such as *cytochrome P450 oxygenase 1B1* (CYP1B1); therefore, we also determined correlations with CYP1B1 expression (Table 2) (18).

**TABLE 1:** TFBIND analysis of TDO2 enhancer.

**Transcription factor**	**Number of sites**	**Mean site similarity score**
AP1	6	0.805
AP2	1	0.796
AP4	2	0.797
CAAT	1	0.983
CAP	3	0.894
CEBPB	1	0.823
COMP1	1	0.809
CREB	3	0.808
CREBP1	1	0.789
GATA1	3	0.882
GATA2	3	0.845
GATA3	1	0.853
MYOD	1	0.813
NFY	2	0.950
OCT1	1	0.829
P53	4	0.856
S8	1	0.829
SP1	1	0.831
STAF	1	0.762
TAXCREB	1	0.751
VMAF	2	0.808

*Transcription factors with binding sites overlapping the −100 to −120 bp human TDO2 enhancer region are essential for constitutive TDO2 expression in T98G cells.*

**TABLE 2:** TDO2-targeting transcription factors with correlations to both TDO2 and CYP1B1, ordered by rank product.

**No.**	**TF**	**Correlation with**		**Rank**
		**TDO2**	**CYP1B1**	**Product**
1	VDR	0.45	0.57	2.2
2	CEBPB	0.46	0.56	3.2
3	MAFB	0.49	0.49	3.2
4	FBN1	0.41	0.56	5.8
5	ELF4	0.43	0.50	7.1
6	STAT6	0.39	0.55	8.0
7	PLEK	0.47	0.38	8.1
8	BNC2	0.40	0.52	8.7
9	PLEK2	0.49	0.30	9.6
10	BATF	0.43	0.43	10.5

These analyses suggested that only *CCAAT/enhancer-binding protein* β (C/EBPβ) was predicted both to bind within the −92 to −130 bp of TDO2 enhancer essential region and to display a strong positive correlation with the expression of TDO2 (Figure 1E, Spearman: 0.47) and CYP1B1 (Figure 1F, Spearman: 0.56, Figure 1G) in TCGA GBM microarray data. We therefore deleted the putative binding motif of C/EBPβ in the TDO2 enhancer (ACCCTGCATCAGCC, −116 to −103 bp) and repeated the measurements of reporter activity (Figure 1H). We found that deletion of the C/EBPβ binding sequence abolished TDO2 reporter activity in T98G cells, indicating that C/EBPβ is responsible for the constitutive TDO2 expression in this cell line. We then experimentally confirmed that C/EBPβ bound the TDO2 enhancer in T98G cells using a chromatin immunoprecipitation (ChIP) assay, showing that C/EBPβ precipitated significantly more TDO2 enhancer than did TDO2 gene body (Figure 1I).

### C/EBPβ and TDO2 Protein Levels Are Correlated in Glioblastoma Samples and Are Associated With Poor Prognosis

**FIGURE 2:**
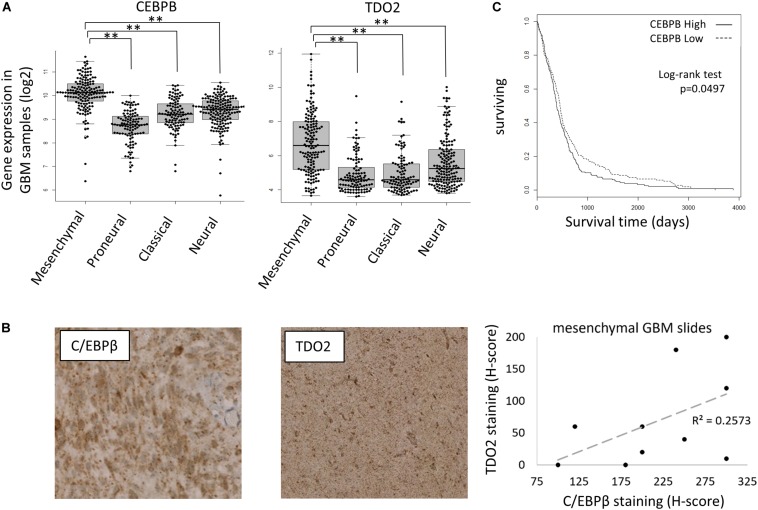
CEBPB expression in glioblastoma (GBM) patients. **(A)** mRNA expression of TDO2 (left) and CEBPB (right) in different GBM subtypes of The Cancer Genome Atlas (TCGA) dataset. **(B)** Immunohistochemical staining confirms a correlation between C/EBPβ and TDO2 protein levels in pathologist-determined mesenchymal subtype GBM tumor sections. **(C)** Kaplan–Meier analysis of overall survival in GBM patients (all subtypes) according to CEBPB mRNA expression (log-rank *p* = 0.0497, high *n* = 263, low *n* = 262). ^∗^*p* < 0.05, ^∗∗^*p* < 0.01, ^∗∗∗^*p* < 0.001, and ^****^p < 0.0001.

C/EBPβ is a member of the *CCAAT/enhancer-binding proteins* (C/EBPs) family of leucine-zipper transcription factors that regulate gene expression controlling inflammation, differentiation, proliferation, and metabolism. In the context of glioma, C/EBPβ was reported as an initiator and master regulator of the GBM mesenchymal transition (28). To confirm that C/EBPβ-mediated expression of TDO2 was not a unique feature of T98G cells, we further examined the TCGA GBM gene expression database, finding both CEBPB and TDO2 to be enriched in the mesenchymal subtype of GBM (Figure 2A). We obtained slides of mesenchymal GBM tumors and confirmed that the correlation between CEBPB/TDO2 held true at the protein level using immunohistochemistry (*R*^2^ = 0.26, Figure 2B). Finally, we examined the effect on patient survival using Cox regression modeling and log-rank testing, finding that GBM expressing high CEBPB showed modestly, but significantly, shorter overall survival in the TCGA dataset (log-rank *p* = 0.0497, Figure 2C).

### CEBPB Expression Is Driven by IL-1β in Glioblastoma

**FIGURE 3:**
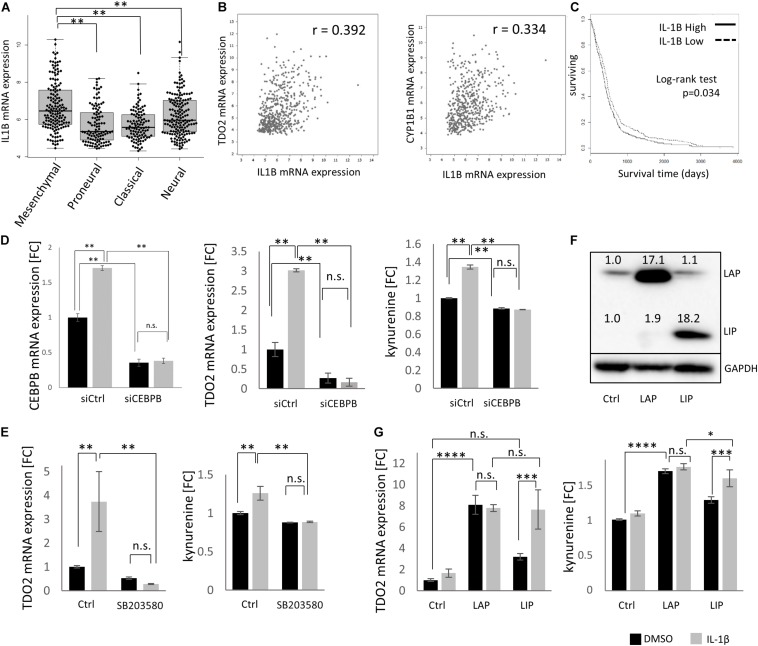
IL1B drives CEBPB expression in glioblastoma (GBM). **(A)** mRNA expression of IL1B in different GBM subtypes within The Cancer Genome Atlas (TCGA dataset. **(B)** mRNA expression of IL1B correlated with TDO2 (left) and CYP1B1 (right) in GBM samples. **(C)** Kaplan–Meier analysis of overall survival of GBM patients according to the expression of IL1B (log-rank *p* = 0.034, high *n* = 263, low *n* = 262). **(D)** siRNA-mediated knockdown of CEBPB in T98G cells results in a decrease of CEBPB mRNA levels, as well as a decrease in TDO2 mRNA expression and concomitant kynurenine (Kyn) levels. **(E)** The CEBPB-driven increase in TDO2 expression (left) and Kyn levels (right) mediated by IL-1β treatment is blocked by the MAPK inhibitor SB203580. **(F)** Stably transfected T98G cells overexpressing CEBPB LIP or LAP isoforms show higher levels of the respective CEBPβ protein isoform. **(G)** TDO2 mRNA expression (left) and Kyn production (right) in T98G cells overexpressing CEBPB LAP or LIP in response to IL-1β treatment. ^∗^*p* < 0.05, ^∗∗^*p* < 0.01, ^∗∗∗^*p* < 0.001, and ^****^p < 0.0001.

As CEBPB is constitutively expressed, with higher levels in mesenchymal GBMs, we next aimed to delineate the signaling events responsible for constitutive CEBPB expression. IL-1β is a key cytokine produced by GBMs that stimulates C/EBPβ transcriptional activity (29, 30). Similar to TDO2 and CEBPB, IL1B was enriched in the mesenchymal subtype of GBM patients in the TCGA dataset (Figure 3A), and as expected, the expression of IL1B positively correlated with TDO2 as well as CYP1B1 in GBM (Figure 3B). Log-rank testing showed that GBM patients with high IL1B expression showed significantly shorter overall survival (Figure 3C, *p* = 0.034), similar to patients expressing high CEBPB (Figure 2C, *p* = 0.0497).

Using T98G cells, we were able to show that TDO2 expression as well as Kyn production was increased by IL-1β treatment and that this effect was completely abolished by C/EBPβ knockdown (Figure 3D). Phosphorylation of C/EBPβ is important for its transcriptional activity, and p38 MAPK is reported to phosphorylate C/EBPβ (31). We confirmed that the IL-1β-induced, C/EBPβ-mediated increase in TDO2 expression and concomitant Kyn production were inhibited by the p38 MAPK inhibitor SB203580 (Figure 3E).

CEBPB mRNA can produce at least three protein isoforms – 38 (LAP^∗^), 35 (LAP), and 20 kDa (LIP) – with the LAP and the LIP forms being the major polypeptides produced in cells (32). Because the LIP protein isoform lacks activation domains, LIP has been thought to repress transcription by competing for C/EBPβ consensus binding sites (33); however, emerging evidence suggests that LIP can also function as a transcriptional activator (34). In T98G cells, only overexpression of the LAP isoform resulted in statistically significant increases in TDO2 mRNA (*p* < 0.0001) and Kyn (*p* < 0.0001), whereas LIP overexpression increased Kyn levels (*p* = 0.0009) and resulted in a non-significant increase in TDO2 mRNA (*p* = 0.0846) (Figures 3F,G). Treatment of CEBPB-transduced T98G cells with IL-1β increased TDO2 expression and Kyn production in LIP overexpressing T98G cells, but not LAP overexpressing cells, suggesting that IL-1β typically induces the LAP isoform and that this had already reached saturating levels in LAP overexpressing T98G cells (Figure 3G).

## Discussion

Solid tumors such as melanoma, breast, lung, and colon cancers constitutively express the Trp degrading enzyme IDO1. TDO2, which is constitutively present in the liver and responsible for regulating systemic Trp levels, has only recently been found to be expressed in cancers and capable of producing Kyn (18–20, 35). In contrast to IDO1, little is known about the regulation of TDO2 in cancer. Previous studies have shown that whereas in hepatocytes TDO2 expression and protein stability is induced by glucocorticoids (36), in GBM cell glucocorticoid signaling suppresses TDO2 expression (37). We have recently demonstrated that prostaglandins, chiefly prostaglandin E2 (PGE2), enhance TDO2 expression and enzymatic activity in malignant gliomas via activation of the prostaglandin E receptor-4 (EP4) (38).

In contrast to previous approaches, here, we used an unbiased search for regulators of constitutive TDO2 expression in a GBM cell line using iteratively deleted TDO2 reporter constructs to show that C/EBPβ plays an important role in driving TDO2 expression in glioma cells. We confirmed our results by using the TCGA database to show strong transcriptional correlations between CEBPB and TDO2 itself, as well as between CEBPB and genes upregulated by TDO2 protein activity such as the AHR target gene CYP1B1 (18). We confirmed that this association holds true at the protein level using immunohistochemistry staining of mesenchymal GBM samples, a GBM subtype in which TDO2 expression is particularly elevated, although in the absence of a tumor cell stain in Figure 2B, we cannot exclude that the CEBP/β staining arises from infiltrating CEBPB-expressing macrophages.

We further showed that CEBPB expression is driven by IL-1β, and we confirmed that CEBPB-mediated upregulation of TDO2 requires the phosphorylation of C/EBPβ, which can be antagonized by the p38 MAPK inhibitor SB203580. Our data suggest that the IL-1β-driven increase in TDO2 expression acts predominantly through the LAP isoform of CEBPB, as previously reported in studies of IL-1β-mediated induction of CEBPB in SW1353 cells (39) and in accordance with the classical view of LAP as a transcriptional activator (32). One caveat to our data was that in some cases we observed a reduced sensitivity to IL-1β in cells held a long time in culture (see low induction of TDO2 mRNA in controls in Figure 3G), although whether this is due to genetic drift at high passage number or other factors is unclear. In line with our interpretation of these data, while preparing this manuscript, Yang and colleagues showed that the livers of mice kept under hypoxic conditions had more of the LAP isoform of CEBPB mRNA as well as more TDO2 protein and that knockdown of CEBPB precluded this increase in TDO2 protein (40).

Our approach demonstrates the value of combining experimental data with database-driven analyses, adding a molecular dimension to the work of Yang and colleagues and uncovering a novel role for this regulatory pathway driving the expression of the TDO2 enzyme in GBM. Given the key role that TDO2 plays in establishing the tryptophan-poor, immunosuppressive tumor microenvironment evident in the mesenchymal subtype of GBM – in which patient prognosis is particularly poor – our work uncovers a potential new target for therapeutic intervention.

## Data Availability Statement

All datasets generated for this study are included in the article/Supplementary Material.

## Author Contributions

TK, MTP, SM, FS, and ZZ generated the data. TK, MP, and EG conceived the experiments and wrote the manuscript. IG, WW, CO, MP, and EG supervised the work.

## Conflict of Interest

The authors declare that the research was conducted in the absence of any commercial or financial relationships that could be construed as a potential conflict of interest.

## Footnotes

1 http://tfbind.hgc.jp

3 http://firebrowse.org/

## Supplementary Material

FIGURE S1 IDO1 and TDO2 expression in TCGA GBM samples. Tryptophan degrading enzymes IDO1 and TDO2 are expressed in a subset of GBM tumors within the TCGA dataset.

TABLE S1 Table of supplementary information regarding experimental materials used in this study, including DNA sequences of primers and transgenic contructs, and antibody clone numbers.
