# Supplementary figures and images for: Constitutive Expression of the Immunosuppressive Tryptophan Dioxygenase TDO2 in Glioblastoma Is Driven by the Transcription Factor C/EBPβ
Source: Front Immunol. 2020 May 14;11:657. doi: 10.3389/fimmu.2020.00657 (PMC7239998; doi:10.3389/fimmu.2020.00657)

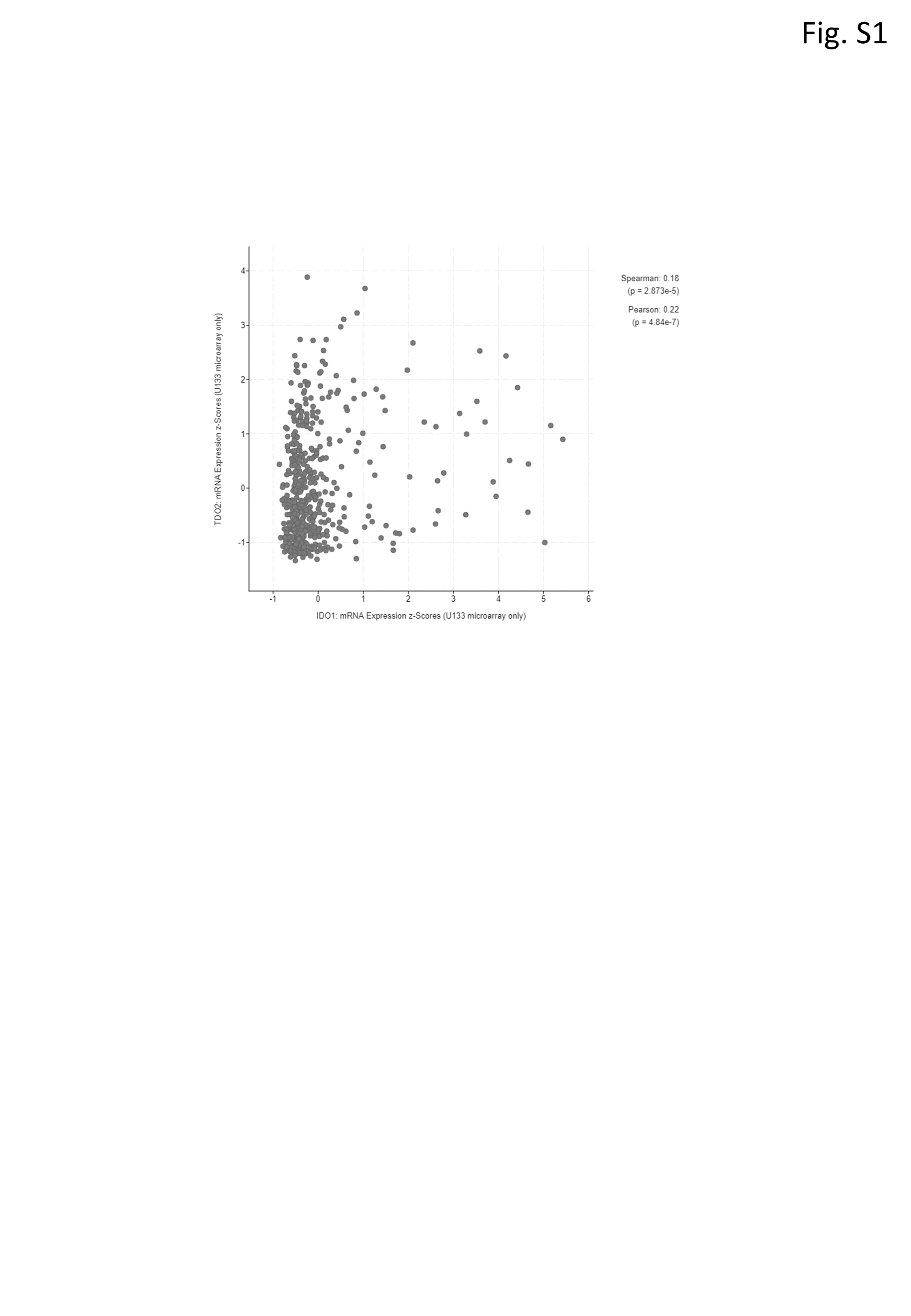

Supplement: FIGURE S1 — IDO1 and TDO2 expression in TCGA GBM samples. Tryptophan degrading enzymes IDO1 and TDO2 are expressed in a subset of GBM tumors within the TCGA dataset. [file Image_1.TIF]
